# Pandemic influenza A (H1N1) virus causes abortive infection of primary human T cells

**DOI:** 10.1080/22221751.2022.2056523

**Published:** 2022-04-25

**Authors:** Jiapei Yu, Hui Li, Ju Jia, Zhisheng Huang, Shuai Liu, Ying Zheng, Shengrui Mu, Xiaoyan Deng, Xiaohui Zou, Yeming Wang, Xiao Shang, Dan Cui, Lixue Huang, Xiaoxuan Feng, William J. Liu, Bin Cao

**Affiliations:** a Department of Basic Medical Sciences, School of Medicine, Tsinghua University, Beijing, People’s Republic of China; b Tsinghua University-Peking University Joint Centre for Life Sciences, Tsinghua University, Beijing, People’s Republic of China; c Department of Pulmonary and Critical Care Medicine, Centre of Respiratory Medicine, China-Japan Friendship Hospital, Beijing, People’s Republic of China; d Laboratory of Clinical Microbiology and Infectious Diseases, China-Japan Friendship Hospital, National Clinical Research Centre for Respiratory Medicine, Beijing, People’s Republic of China; e Institute of Respiratory Medicine, Chinese Academy of Medical Sciences and Peking Union Medical College, Beijing, People’s Republic of China; f Department of Pulmonary and Critical Care Medicine, Clinical Centre for Pulmonary Infections, Capital Medical University, Beijing, People’s Republic of China; g Department of Respiratory and Critical Care Medicine, Shandong Provincial Hospital Affiliated to Shandong First Medical University, Jinan, People’s Republic of China; h Department of Respiratory Medicine, Harbin Medical University, Harbin, People’s Republic of China; i Department of Respiratory Intensive Care Unit, The First Affiliated Hospital of Zhengzhou University, Zhengzhou, People’s Republic of China; j NHC Key Laboratory of Biosafety, Chinese Centre for Disease Control and Prevention, National Institute for Viral Disease Control and Prevention, Beijing, People’s Republic of China

**Keywords:** Influenza A virus, single-cell sequencing, primary T cells, abortive infection, virus-host interactions

## Abstract

Influenza A virus still represents a noticeable epidemic risk to international public health at present, despite the extensive use of vaccines and anti-viral drugs. In the fight against pathogens, the immune defence lines consisting of diverse lymphocytes are indispensable for humans. However, the role of virus infection of lymphocytes and subsequent abnormal immune cell death remains to be explored. Different T cell subpopulations have distinct characterizations and functions, and we reveal the high heterogeneity of susceptibility to viral infection and biological responses such as apoptosis in various CD4^+^ T and CD8^+^ T cell subsets through single-cell transcriptome analyses. Effector memory CD8^+^ T cells (CD8^+^ T_EM_) that mediate protective memory are identified as the most susceptible subset to pandemic influenza A virus infection among primary human T cells. Non-productive infection is established in CD8^+^ T_EM_ and naïve CD8^+^ T cells, which indicate the mechanism of intracellular antiviral activities for inhibition of virus replication such as abnormal viral splicing efficiency, incomplete life cycles and up-regulation of interferon-stimulated genes in human T cells. These findings provide insights into understanding lymphopenia and the infectious mechanisms of pandemic influenza A virus and broad immune host–pathogen interactional atlas in primary human T cells.

## Introduction

Influenza is a common respiratory disease caused mainly by the influenza A virus, which is a single-strand RNA virus with negative-sense segmented genome [1]. Although it is characterized by seasonal epidemics every year, unpredictable emergencies of global pandemic still appear at intervals such as the Spanish flu (1918), Asian flu (1957), Hong Kong flu (1968) and swine flu (2009) [2]. Patients infected with influenza virus are characterized by lymphopenia. And it’s worth noting that severe lymphopenia is a key independent risk factor of worse clinical outcomes in hospitalized patients with influenza pneumonia [3]. The previous study further indicated that the lower levels of absolute counts of CD4^+^ T and CD8^+^ T cells in peripheral blood had been proposed to be a marker correlated with death [4]. However, the pathological mechanism of lymphopenia is still not quite clear.

T cells are essential in the human adaptive immune response against influenza infection. Persistent alterations in function and subset composition of T cells post-infection have momentous implications for the long-term prognosis of patients. CD4^+^ T cells secrete a series of cytokines and help with the production of protective antibodies, while CD8^+^ T cells recognize endogenous antigen presented on MHC I (major histocompatibility complex I) by antigen-presenting cells and release cytotoxic granules to kill infected cells. In addition, immunological memory of CD4^+^ and CD8^+^ T cells is induced upon infection, which is primarily directed to conserved viral antigen peptides and formulate cross-reactivity with different subtypes in influenza A virus reinfection. In T cell adaptive system, reactive memory is regulated mainly by central memory T cells (T_CM_) with differentiation into effector cells and proliferation post-stimulation at high speed, and protective memory is mediated through effector memory T cells (T_EM_) which migrate to infection sites and perform rapid effector function nearby [5].

The immune system mediated by T lymphocytes has become so crucial that the role of virus infection of lymphocytes and subsequent abnormal immune cell death remains to be explored. It has been reported that influenza A virus can infect almost all types of human innate immune cells directly with corresponding pathological modifications, such as monocytes [6], macrophages [7], DC (dendritic cells) [8], mast cells [9], neutrophils [10] and NK (natural killer) cells [11]. Most of them are non-productive infections with the exception of macrophages and neutrophils [12]. For instance, although there is little biosynthetic activity due to the deficiency of endoplasmic reticulum and ribosomes [13], neutrophils can still produce and release infectious progeny. And interestingly enough, the H1N1 virus (A/Nanchang/8002/2009) can enter into neutrophils through multiple endocytosis in the absence of α-2,3- and α-2,6-linked sialic acid receptors on the cell surface [12].

For adaptive immune cells, B cells in the lung of mice were infected by H1N1 indirectly by taking advantage of interactions between BCR (B cell receptors) and HA (haemagglutinin) [14]. In addition, some studies have shown that the influenza A virus can infect T cells. In an H5N1 infected patient, viral HA and NP (nucleoprotein) could be found in T cells of hilar lymph nodes [15]. What’s more, approximately 10% of primary mice CD3^+^ T cells from the thymus and spleen were found to contain viral HA protein post-infection *in vitro* [16]. In H1N1-treated mice, viral RNAs were detected in around 22.2% of T cells from lung tissue, and the rate of infection is comparable to monocytes/macrophages (25.7%), NK (26.2%) and B cells (31.0%) [17]. The details and influences of direct infection of human T cells by influenza A virus still remains undetermined.

To study human T cell responses to influenza A virus, we investigated whether or not pandemic H1N1 infects human T cells and how pandemic H1N1 infection proceeds. In this research, we further evaluate the heterogeneity of viral infection and host responses in different human T cell subpopulations through single-cell sequencing. Most of all, effector memory CD8^+^ T cells (CD8^+^ T_EM_) are an especially susceptible subset to pandemic H1N1 infection among total T cells, and it may be related to the higher expression of α-2,6-linked sialic acid receptors. In addition, H1N1 infection of T cells did not induce further differentiation. Up-regulation of ISG and MHC I-immunoproteasomes constitutes intracellular antiviral activities and results in non-productive infection.

## Materials and methods

### Cell culture

MDCK cells and A549 cells were gifts from William J. Liu Lab (Chinese Centre for Disease Control and Prevention) and MDCK.2 cell line was purchased from ATCC. Both of them were cultured in DMEM (Dulbecco’s modified Eagle’s medium) supplemented with high glucose and L-glutamine (Gibco®) in addition to 10% FBS (fetal bovine serum) and 1% penicillin/streptomycin in a 5% CO_2_ incubator at 37°C. The cell lines used are routinely tested for mycoplasma and are maintained mycoplasma-free. PBMCs (peripheral blood mononuclear cells) were isolated from fresh whole blood with anticoagulant of EDTA-K3 through the gradient centrifugation method. Immune cells such as CD14^+^ mononuclear/macrophages, CD4^+^ T cells, CD8^+^ T cells, CD8^+^ T_CM_, CD8^+^ T_EM_ and naïve CD8^+^ T cells were purified from fresh PBMCs through immunomagnetic selection using corresponding EasySep™ magnetic beads separation kit (Stemcell®). The purities of all immune cells are greater than 95% for experiments. All of T cells were cultured in a commercial ImmunoCult™-XF T cell expansion medium (Stemcell®) which was optimized for the in vitro culture and expansion of human T cells isolated from peripheral blood in a 5% CO_2_ incubator at 37°C.

### Virus preparation and infection

The pandemic influenza A virus original strain used in this research was H1N1 (A/California/07/2009) which was gift-giving by William J. Liu Lab (Chinese Centre for Disease Control and Prevention). All laboratory procedures involving live viruses were performed in a biosafety level 2 (BSL-2) facility. The influenza viruses were cultured and propagated on MDCK (Madin-Darby canine kidney) cells with specialized serum-free medium for influenza virus isolation (Yocon biology, NC0202) and serum-free medium for MDCK cells culture (Yocon biology, NC0201), and tittered by TCID_50_ through the Reed-Muench method. For live influenza virus infection, purified fresh primary CD8^+^ T_EM_ and naïve CD8^+^ T cells were incubated with the indicated viral strain at a MOI (multiplicity of infection) of 10 for 1 h at 37°C and then washed with PBS adequately. For inactivated viral treatment of different T cells, influenza virus was first inactivated by UV for 30 min.

### Single-cell sequencing

CD4^+^ and CD8^+^ T cellular suspensions were loaded on the 10× Genomics GemCode Single-cell instrument which generated single-cell Gel Bead-In-EMlusion (GEMs). Libraries were generated and sequenced from the cDNAs with Chromium Next GEM Single Cell 3′ Reagent Kits v3.1. Upon dissolution of the Gel Bead in a GEM, primers containing: an Illumina® R1 sequence (read 1 sequencing primer), a 10 nt UMI (unique molecular identifier), a 16 nt 10× Barcode, and a poly-dT primer sequence were released and mixed with cell lysate and Master Mix. Barcoded, full-length cDNAs were then reverse-transcribed from poly-adenylated mRNA.

To identify single-cells with viral RNA, we aligned raw scRNA-seq reads using kallisto/bustools (KB) against a customized reference genome, in which the genome of A/California/07/2009 (H1N1):

**Table:** 

name	location	aliases
*HA*	NC_026433.1	UJ99_s4gp1
*NA*	NC_026434.1	UJ99_s6gp1
*PA*	NC_026437.1	UJ99_s3gp1
*PA-X*	NC_026437.1	UJ99_s3gp2
*PB1*	NC_026435.1	UJ99_s2gp1
*PB2*	NC_026438.1	UJ99_s1gp1
*M1*	NC_026431.1	UJ99_s7gp2
*M2*	NC_026431.1	UJ99_s7gp1
*NS1*	NC_026432.1	UJ99_s8gp2
*NEP(NS2)*	NC_026432.1	UJ99_s8gp1

from NCBI Ref.seq was added as an additional chromosome to the human reference genome. Single cell with viral reads (UMI > 0) was retained as infected cell. Cells with less than 200 genes expressed or more than 10% mitochondrial counts were excluded, as well as those labelled as doublet following aforementioned protocol. Bioinformatics analysis of single-cell sequencing data were performed using the OmicShare tools, a free online platform for data analysis (https://www.omicshare.com/tools).

### RNA extraction and RT–PCR

The total RNAs of different samples were extracted with Qiagen RNeasy Mini Kit (74104). RNA quantity and quality were assayed using a Nanodrop 2000 spectrophotometer (Thermo-Fisher). Two-step method was used for qPCR. RNA samples were then converted to cDNA through reverse transcription using an Thermo Scientific™ RevertAid First Strand cDNA Synthesis Kit (K1622). Once cDNA samples were made, qPCR tests were run on an Applied Biosystems QuantStudio® 12K Flex Real Time PCR thermocycler (Life Technologies™) with Forget-Me-Not™ qPCR Master Mix (Biotum EvaGreen®, 31042-1) and using influenza viral:

**Table:** 

	F (5’-3’)	R (5’-3’)
HA	GTATAGGTTATCATGCGAACA	CTCTGGATTTCCCAGGATC
NP	TCAGTGATTATGATGGACGACTA	GCACTGGGATGCTCTTCTAGGTA
M1	TGCTGATTCACAGCATCGG	TGTTCCATAGCCTTTGCCG
M2	GAGGTCGAAACGCCTAC	CTGTTCCTGTTGATATTCTTCCC
NS1	AAAGGAAGAGGCAACACCCT	CCTCGAGGGTCATGTCAGAA
NEP(NS2)	GCTTTCAGGACATACTTATGAGGA	CTCTCGCCACTTTTCATTTCT

And human:

**Table:** 

	F (5’-3’)	R (5’-3’)
rsad2	CGTCAACTATCACTTCACTCG	AATCCTCTCTTTGCTTCCTCA
adar1	TCCGTCTCCTGTCCAAAGAAGG	TTCTTGCTGGGAGCACTCACAC
oas1	AGGAAAGGTGCTTCCGAGGTAG	GGACTGAGGAAGACAACCAGGT
oas2	GCTTCCGACAATCAACAGCCAAG	CTTGACGATTTTGTGCCGCTCG
ns1-bp	GGAGACAGTCTGGAAGAGCTGA	CATCACTGCCAAACACCTCAGC
sf2	TATCCGCGACATCGACCTCAAG	AAACTCCACCCGCAGACGGTAC
hnRNP-K	GCAGATGGCTTATGAACCACAGG	AATCCGCTGACCACCTTTGCCA
β-actin	GTACGCCAACACAGTGCTG	CGTCATACTCCTGCTTGCTG

primers (5′ → 3′). ΔCt method was used to analyse viral splicing ratios as other research group [18]. Quantifications and analyses were done through 2^-ΔΔCt^ method and using Applied Biosystems QuantStudio® 12K software (Life Technologies™, version 1.2.3).

and Canis:

**Table:** 

	F (5’-3’)	R (5’-3’)
ACTB	TTCCGCTGCCCAGAGGCTCT	GCTCAGGGGGTGCGATGATCTTG

### Immunofluorescence staining

After suspension culture for 16h, influenza virus infected CD8^+^ T_EM_ and naïve CD8^+^ T cells were washed with PBS and then fixed in 4% PFA (paraformaldehyde) for 20 min in 1.5 mL microtube, followed by simultaneous permeabilization and blocking with blocking buffer consisting of 1% triton X-100 in 5% BSA (w/v) in PBS for 1 h at room temperature. Primary antibodies to α-2,6-linked sialic acid receptors (Vector Laboratories®, FL-1301-2), CD45RO (abcam, [UCH-L1] ab23) and influenza H1N1 viral HA antibody (Sino Biological®, 11085-T54) was diluted 1:200 in blocking buffer (1% BSA) and incubated overnight at 4 °C. Cells were later washed three times in PBS (allowing 5 min per wash) and labelled with secondary antibodies conjugated to Alexa-594 (abcam, ab150116) and Alexa-647 (abcam, ab150079) respectively for 2 h at room temperature in 1% BSA. α-2,6-linked sialic acid receptors can colour directly without help of secondary antibody. Nuclei were stained with DAPI (4',6-diamidino-2-phenylindole) followed by washing three times. Images were obtained with a LSM 880 inverted confocal microscope (Carl Zeiss, Jena, Germany).

### Flow cytometry analysis

For surface marker staining, T cells were washed with PBS, and then resuspended in eBioscience™ flow cytometry staining buffer (Invitrogen, 00-4222-57). Cell suspension was filtered with 0.45 μm filters, and incubated with antibodies of BV605-conjugated anti-CD4, BV421-conjugated anti-CD8, redFluor™ 710-conjugated anti-CD14, APC-conjugated anti-CD45RA, PE-conjugated anti-CD45RO, Alexa Fluor® 647-conjugated anti-CD62L, Alexa Fluor® 700-conjugated anti-CCR7 (CD197). Flow cytometry was performed using CytoFLEX LX V5-B4-R3 instruments (Beckman Coulter Life Sciences, 12 detectors, 3 lasers). Gating was performed to remove cellular debris and to ensure analysis was performed only on singlet cells as determined by forward and side scatter measurements. Data were analysed using FlowJo software (version 8.1.2).

## Results

### Highly heterogeneity exists in the ability of pH1N1 to infect different subsets of primary human T cells and CD8+ TEM is relatively more susceptible

Human T cells differentiate into heterogeneous populations of effector or memory T cells that can help pathogen clearance after infection. We hypothesized that primary human T cells were potential targets of pandemic H1N1 infection according to the existing experimental conclusion [19]. The pure human CD4^+^ and CD8^+^ T cells were incubated with H1N1 (A/California/07/2009) at a MOI (multiplicity of infection) of 10 for 1h *in vitro* respectively. After washing with PBS, the cells were collected immediately as the 0 h.p.i. group while the others were collected after a culture of 16 h. Together with the controls, all samples were prepared as single-cell suspensions for 10× Genomics sequencing. The transcription process of influenza A virus is special that the viral mRNA with 3′-poly(A) tail have to be in the nucleus when they are produced, and that’s exactly what the single-cell sequencing detects. So, it can tell us very precise information about “infection” rather than “contamination” (false positive) due to the lack of adequate washing.

**Figure 1.**
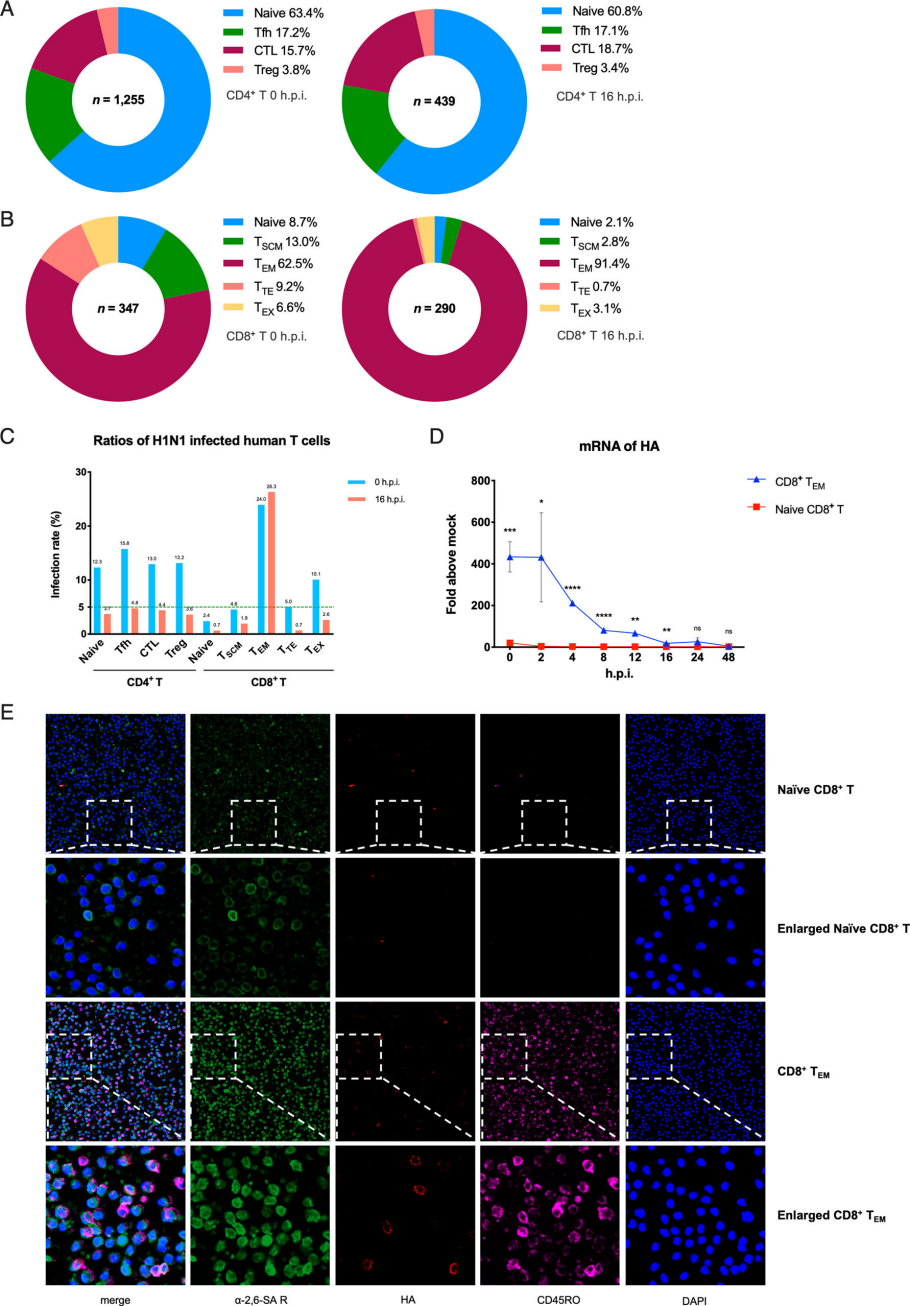
H1N1 infected different subsets of primary human T cells heterogeneously. (A) Distribution of different subsets in infected human CD4^+^ T cells. Left, 0 h.p.i.; right, 16 h.p.i. Blue, naïve CD4^+^ T; green, follicular helper T (Tfh); wine, CD4^+^ cytotoxic T (CTL); rose pink, regulatory T (Treg). (B) Distribution of different subsets in infected human CD8^+^ T cells. Left, 0 h.p.i.; right, 16 h.p.i. Blue, naïve CD8^+^ T; green, stem cell memory T (T_SCM_); wine, effector memory T (T_EM_); rose pink, terminally differentiated effector T (T_TE_); yellow, exhaust T (T_EX_). (C) Infection rates among different primary human CD4^+^ and CD8^+^ T subsets. Blue, 0 h.p.i.; red, 16 h.p.i.; green dotted line, 5%. (D) mRNA expression levels of viral HA in naïve CD8^+^ T and CD8^+^ T_EM_ from one healthy donor at eight time points. Results are represented as mean fold change ± SD and statistical significances were analysed using GraphPad Prism 8.0 through Student’s *t*-test. *p < 0.05, **p < 0.01, ***p < 0.001, ****p < 0.0001, ns = non-significant. (E) Immunofluorescent staining of naïve CD8^+^ T and CD8^+^ T_EM_ which exposure to H1N1 respectively. Green, α-2,6-linked sialic acid receptors; pink, CD45RO; red, viral HA proteins; blue, DAPI (4′,6-diamidino-2-phenylindole).

The results showed highly heterogeneity of viral infection in different T subsets. Firstly, effector memory CD8^+^ T cells (CD8^+^ T_EM_) is the most susceptible T cell subset with 24.0% (0 h.p.i.) and 26.3% (16 h.p.i.) proportion of infection in its own subpopulation. In particular, the infection rate of CD8^+^ T_EM_ was 4.5 times higher than CD4^+^ Tfh cells and 36.5 times higher than naive CD8^+^ cells at 16 h.p.i., and all other T subsets are less than 5% at 16 h.p.i. (Figure 1C). Besides, more than 90% of the infected CD8^+^ T cells were CD8^+^ T_EM_ after 16 h surprisingly (Figure 1B). Secondly, the rate of naïve CD8^+^ T cells in total infected CD8^+^ T cells was less than 10%. In contrast to that, naïve CD4^+^ T cells are the most susceptible subset among CD4^+^ T cells, which accounts for more than 60% of all the infected CD4^+^ T cells at both two different points (Figure 1A). What’s more, for total CD4^+^ T cells, infection rate decreases somewhat after 16 h while it remains essentially unchanged in total CD8^+^ T cells (Figure S1A).

To further characterize the heterogeneity of influenza A virus infection in T cells, we then challenged two major subsets of human CD8^+^ T cells, CD8^+^ T_EM_ and naïve CD8^+^ T with H1N1 and checked for the expression of viral HA via qPCR. The mRNA level of HA in CD8^+^ T_EM_ was about 200–400 times higher than naïve CD8^+^ T cells at 0–16 h.p.i. points in 48 h over time (Figure 1D). In addition, immunofluorescent staining also indicated that HA proteins seem to be gathered on the cell sections of CD8^+^ T_EM_ rather than naïve CD8^+^ T cells more intensively (Figure 1E). In conclusion, H1N1 showed more powerful infection ability in CD8^+^ T_EM_ than other human T cell subsets.

### The main cause for higher susceptibility of human CD8^+^ T_EM_ to H1N1

It is well-known that the sialic acid receptor is the most primary receptor for the viral entry process into host cells, which is recognized and bound by viral HA membrane proteins [20]. α-2,6-linked sialic acid receptors are abundant in the upper respiratory tract of humans and bind to human influenza viruses predominantly, while α-2,3- are rich in human lower respiratory tract and recognize avian influenza viruses preferentially [21]. Sialic acid linkages are cell type-specific and change on the cell surface accompanying with differentiation and activation of T cells. Therefore, the distribution and type of sialic acid receptor expression might be a crucial determinant of influenza A virus’s tropism of different T cells [22]. We next investigated the quantitative expression of sialic acid receptors on different primary human T subsets.

**Figure 2.**
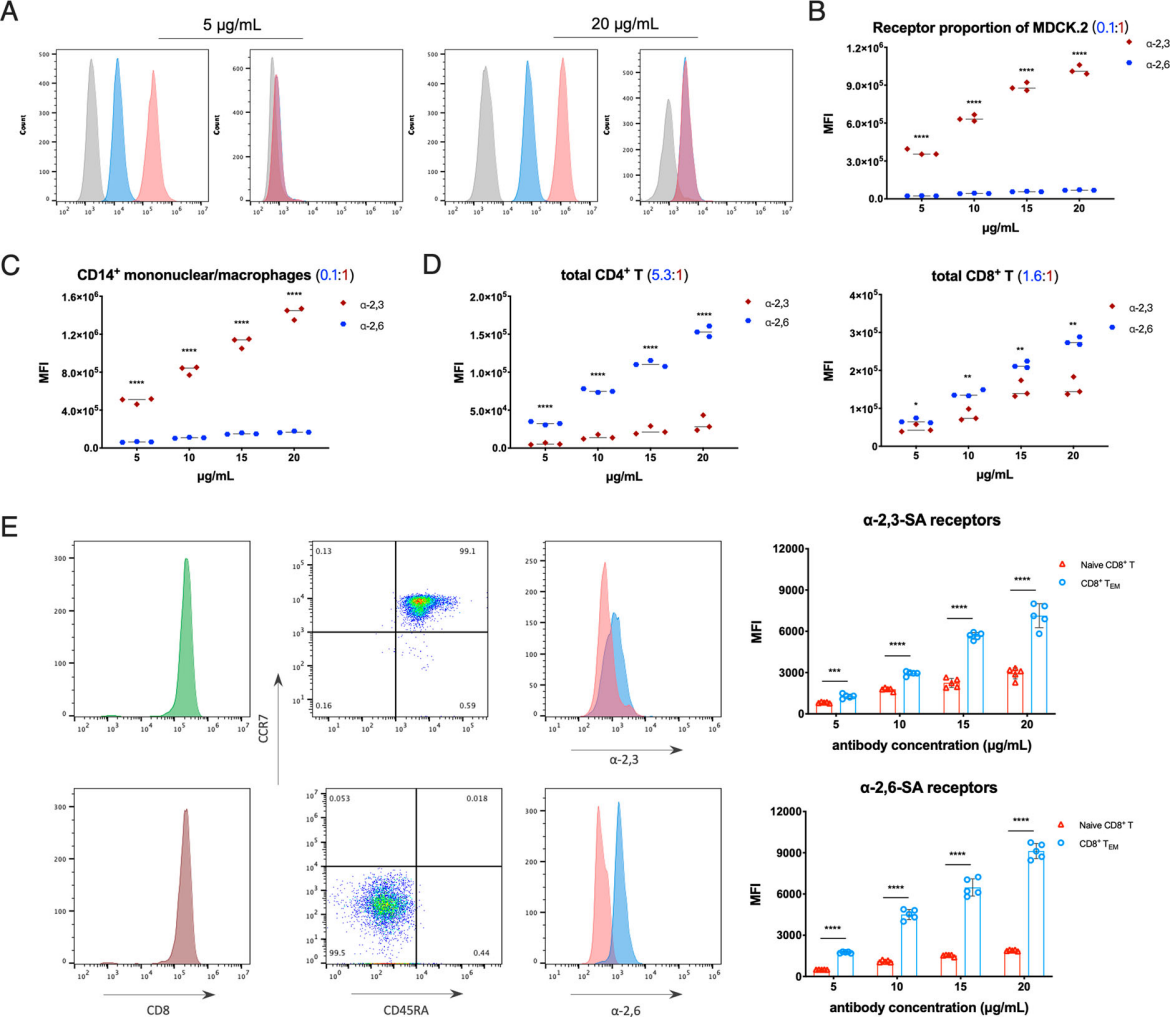
Quantitative analysis of α-2,3- and α-2,6-linked sialic acid receptors in MDCK.2 and primary human T cells. (A) Flow cytometry analysis of broad-spectrum neuraminidases treated MDCK.2 cells or not with the lectin antibody concentrations of 5 and 20 μg/mL. Grey, unstained as controls; blue, α-2,6-linked sialic acid receptors; red, α-2,3-linked sialic acid receptors. (B) The comparison of α-2,6- and α-2,3-linked receptors in MDCK.2 cells from ATCC. (C) The comparison of α-2,6- and α-2,3-linked receptors of human CD14^+^ mononuclear/macrophages. (D) The comparison of α-2,6- and α-2,3-linked receptors of human total CD4^+^ T and total CD8^+^ T cells. (E) Purities of gating and MFI of receptors with the lectin antibody concentrations of 5 μg/mL in CD8^+^ T_EM_ and naïve CD8^+^ T cells and the quantitative analysis of α-2,6- and α-2,3-linked sialic acid receptors between CD8^+^ T_EM_ and naïve CD8^+^ T cells from five healthy donors. Both CD8^+^ T_EM_ and naïve CD8^+^ T cells were stained with identical fluorescent antibodies. Red, naïve CD8^+^ T; blue, CD8^+^ T_EM_. Results of (B-E) are represented as mean fold change ± SD and statistical significances were analysed using GraphPad Prism 8.0 through two-tailed Student’s *t*-test. *p < 0.05, **p < 0.01, ***p < 0.001, ****p < 0.0001.

Two FITC-conjugated phytolectins were used in the study, *Sambucus nigra* bark lectin (SNA) binds to α-2,6-linked sialic acid receptors and *Maackia amurensis* lectin (MAL) binds to α-2,3-linked sialic acid receptors, respectively [23]. MDCK.2 cells were chosen as control which expresses α-2,3- and α-2,6-linked sialic acid receptors undeniably, and we also treated MDCK.2 cells with broad-spectrum neuraminidases to cleave all surface sialic acid residues and confirmed the specificity of SNA and MAL staining at gradient working concentrations (Figure 2A).

The sialic acid receptors ratio of α-2,6- to α-2,3- in MDCK.2 cells is around 0.1:1 through MFI (mean fluorescence intensity) data from flow cytometry analyses (Figure 2B). For immune cells, the fraction of CD14^+^ mononuclear/macrophages was similar to MDCK.2 cells as 0.1:1 (Figure 2C), while CD4^+^ T cells showed higher α-2,6- than α-2,3- receptors with a ratio of 5.3:1 (Figure 2D). In terms of total human CD8^+^ T cells, α-2,6- was relatively close to α-2,3-linked with a ratio of 1.6:1 (Figure 2D). And it is interesting that the rises in the proportion of α-2,6- to α-2,3-linked receptors were detected in each subset on the differentiation process of CD8^+^ T cells: naïve T is ∼ 0.7:1, T_EM_ is ∼ 1.3:1 and T_CM_ is ∼ 4:1 (Figure S1D). Taken together, the data suggested that both α-2,3- and α-2,6-linked sialic acid receptors were expressed on the surface of human T cell subsets, but with different type ratios. Further, there was a huge distinction between CD8^+^ T_EM_ and other T cell subsets concerning the expression quantity of sialic acid receptors. To ensure the comparability of results, identical fluorescent antibodies were used on isolated CD8^+^ T_EM_ and naïve CD8^+^ T cells. More importantly, α-2,6-linked sialic acid receptors on the surface of CD8^+^ T_EM_ were four times more than naïve CD8^+^ T cells (Figure 2E and S1D). Anisotropic amount of the receptors between CD8^+^ T_EM_ and naïve CD8^+^ T cells may be the main cause of susceptibility to viral infection.

### Non-productive infection in primary human CD8^+^ T_EM_

Influenza A virus has its unique life cycle in host cells. A successful replication mainly depends on host cells to provide organelles such as endoplasmic reticulum, dictyosomes, mitochondria and ribosomes, until the intact infectious progeny virus is released from original cells favourably. However, sometimes there is no production of new virions due to the virus cannot hijack the host cell completely, such as the abortive infection of macrophages and NK cells for most seasonal influenza A strains [11,24]. So, we explored whether the infected human T cells could produce progeny viruses or not.

**Figure 3.**
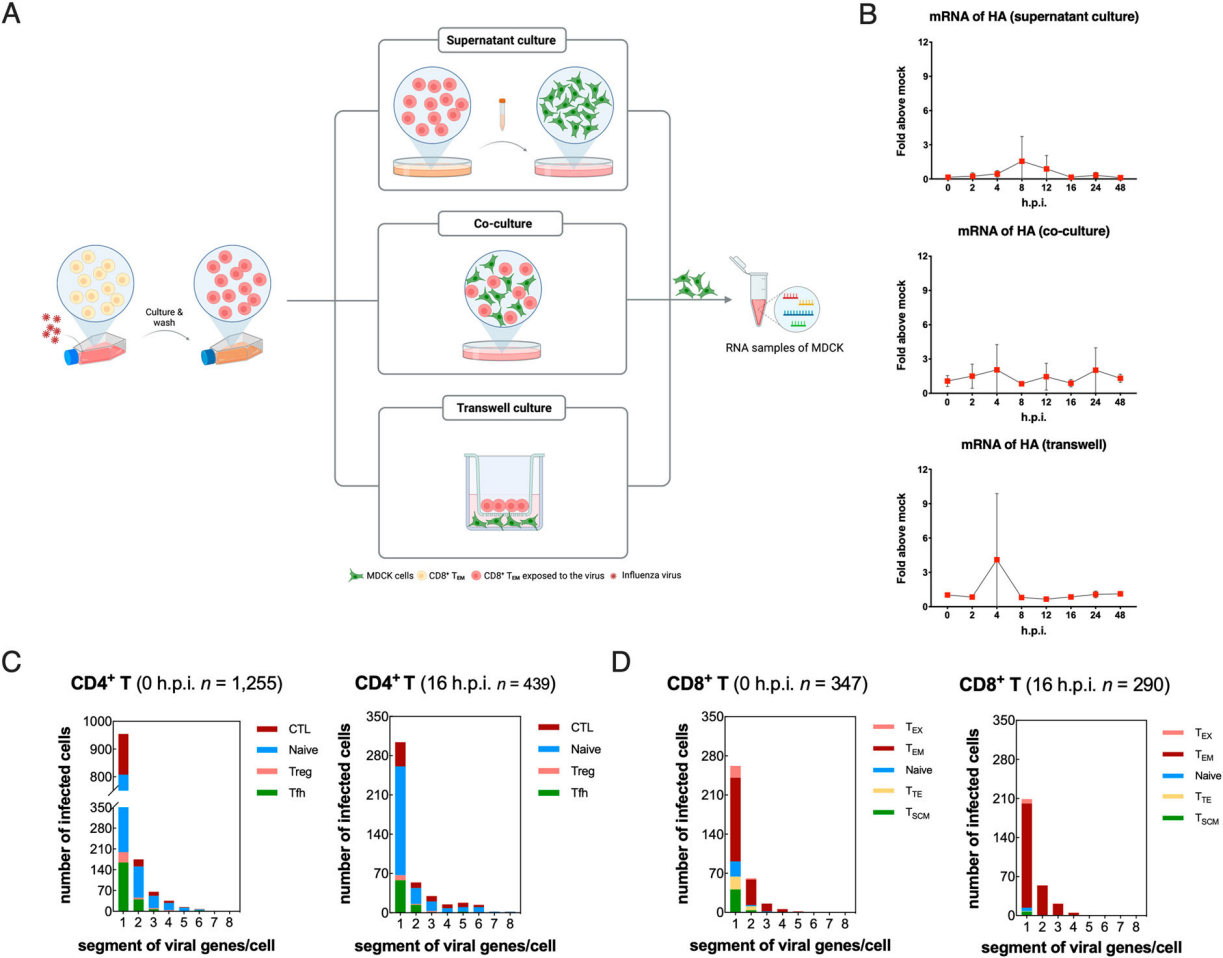
Abortive infection of pandemic H1N1 was established in primary human T cells. (A) Schematic diagram of supernatant culture, transwell culture and co-culture directly between infected CD8^+^ T_EM_ and untreated MDCK cells. (B) mRNA expression levels of viral HA in samples of MDCK cells among the above three culture methods. Results are represented as mean fold change ± SD and statistical significances were analysed using GraphPad Prism 8.0 through two-tailed Student’s *t*-test. *p < 0.05, ns = non-significant. (C) Numbers of H1N1 viral genes per cell in different infected CD4^+^ T subsets. Blue, naïve CD4^+^ T; green, follicular helper T (Tfh); wine, CD4^+^ cytotoxic T (CTL); rose pink, regulatory T (Treg). (D) Numbers of H1N1 viral genes per cell in different infected CD8^+^ T subsets. Blue, naïve CD8^+^ T; green, stem cell memory T (T_SCM_); wine, effector memory T (T_EM_); rose pink, terminally differentiated effector T (T_TE_); yellow, exhaust T (T_EX_).

In terms of infected human CD8^+^ T_EM_, the mRNA expression levels of viral NP gene also tended to decrease post-infection just like HA gene (Figure S1B and S1C). To further clarify whether H1N1 could replicate in CD8 T_EM_ to produce live viruses, we performed co-culture of infected CD8^+^ T_EM_ with MDCK cells *in vitro*. The washed infected CD8^+^ T_EM_ was cultured for 16 h and the supernatant was adsorbed with MDCK cells as the first group. Second, infected CD8^+^ T_EM_ was cultured with MDCK in transwell devices that share the same medium but do not contact directly. In the third group, adherent MDCK was co-cultured with suspended infected CD8^+^ T_EM_ with a relationship of immediate contact (Figure 3A). There was almost no detectable level of viral HA mRNA in MDCK cells among all three groups, which indicated that the abortive infection in the most susceptible T subset with no release of infectious progeny virions (Figure 3B). Further, we found that in infected human T cells, complete eight mRNA gene fragments of the influenza virus could not be detected at the same time, but only up to 5∼6 mRNA gene fragments could be detected in one cell. In addition, 96.5% of infected CD4+ T cells and 99.7% of infected CD8^+^ T cells contained no more than four viral gene segments, indicated that infectious progeny of H1N1 could not be replicated in and released from human T cells.

### The main cause for abortive infection in primary human T cells

**Figure 4.**
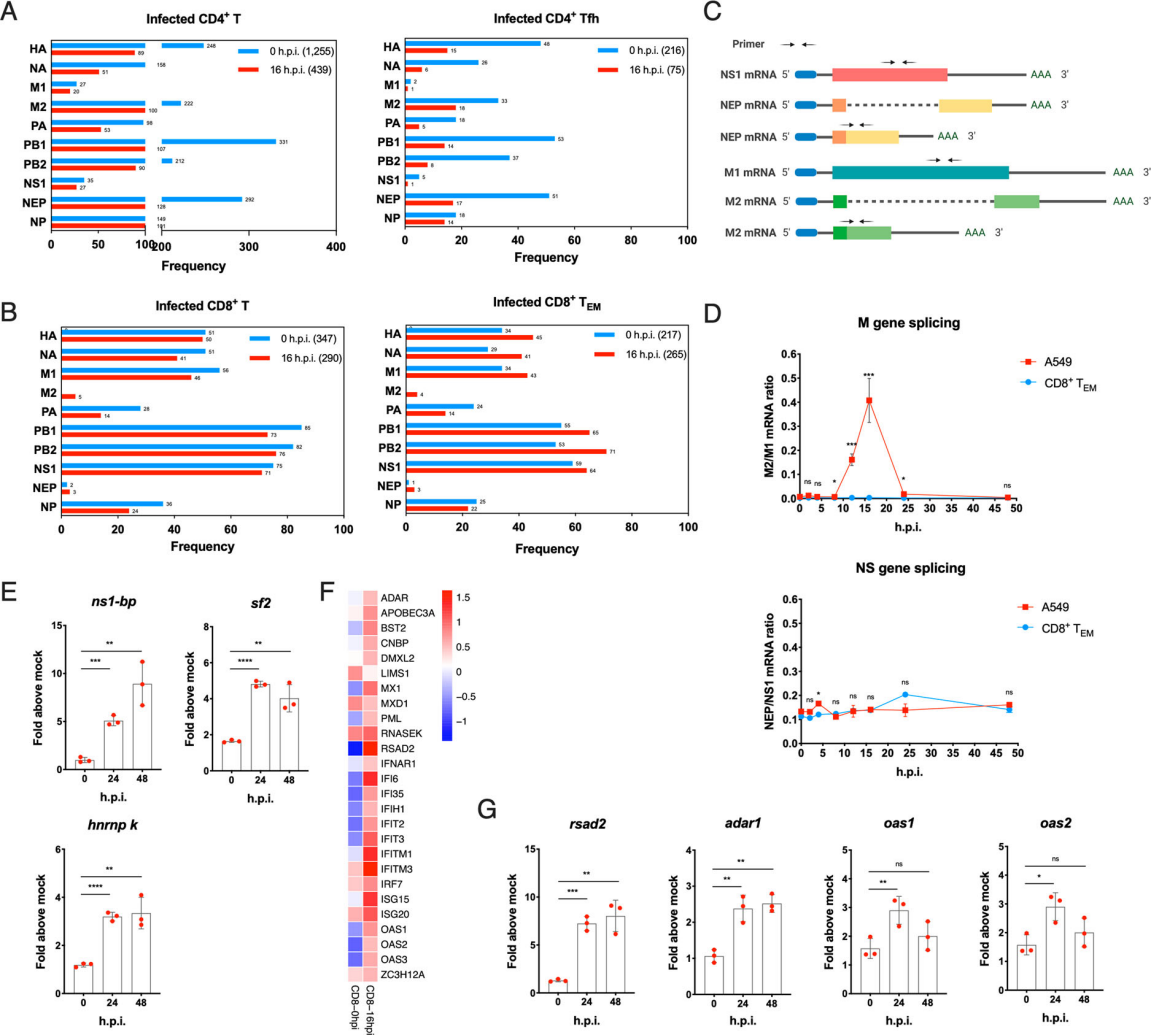
Abnormal splicing efficiency and up-regulation of ISGs dedicate to work for abortive infection in primary human T cells. (A) Frequency of different H1N1 viral genes in infected total CD4^+^ T and follicular helper T cells. Blue, 0h.p.i.; red, 16 h.p.i. (B) Frequency of different H1N1 viral genes in infected total CD8^+^ T and CD8^+^ T_EM_. Blue, 0h.p.i.; red, 16 h.p.i. (C) Schematic representation of alternative splicing of M1 and NS1 mRNA and their alternatively spliced product M2 and NEP (NS2) mRNA. The arrowheads show corresponding primer positions for detection. (D) Alternative splicing efficiency of M and NS genes in infected CD8^+^ T_EM_ and A549 cells post-infection. Red, A549 cells; blue, CD8^+^ T_EM_. Results are represented as mean fold change ± SD and statistical significances were analysed using GraphPad Prism 8.0 by two-tailed Student’s *t*-test. *p < 0.05, **p < 0.01, ***p < 0.001, ****p < 0.0001, ns = non-significant. (E) mRNA expression levels of cell host splicing factors *ns1-bp*, *sf2* and *hnRNP K* in CD8^+^ T_EM_ post-infection. (F) The gene heatmap of ISGs in infected CD8^+^ T_EM_. Blue, down-regulation; red, up-regulation. (G) mRNA expression levels of *rsad2*, *adar1*, *oas1* and *oas2* in CD8^+^ T_EM_ post-infection. Results of (E and G) are represented as mean fold change ± SD and statistical significances were analysed using GraphPad Prism 8.0 by two-tailed Student’s *t*-test. *p < 0.05, **p < 0.01, ***p < 0.001, ****p < 0.0001, ns = non-significant.

Although key factors leading to productive infection in epithelial cells of influenza A virus are well defined, there is still limited knowledge about factors leading to the abortive life cycle in human T cells. The host splicing mechanisms play a very critical role in replication of influenza A virus [25]. Viral M and NS genes express unspliced and spliced transcripts for different viral proteins, which is elaborately regulated. In total infected CD4^+^ T cells, both M1 and NS1 mRNA were at extremely low frequency compared to their respective spliced transcripts M2 and NEP. For 0 h.p.i., there were 249 M gene mRNAs, of which M1 transcripts accounted for only 10.8%; at 16 h.p.i., there were 120 M gene mRNAs, of which M1 transcripts accounted for 16.7%. And unspliced NS1 gene RNA was 75, 71 respectively at 0 and 16 h.p.i, whereas its spliced transcript was ten times lower. Similar results were observed in subsets of CD4^+^ T cells. The above results indicate that in CD4^+^ T cells, H1N1 virus is likely to have excessive splicing efficiency after infection (Figure 4A). On the contrary, both M2 and NEP mRNA were significantly lower than the unspliced transcripts M1 and NS1 in total infected CD8^+^ T cells, and it also applied to each CD8^+^ T subsets, especially CD8^+^ T_EM_ (Figure 4B). For instance, the most susceptible CD8^+^ T_EM_ had 34 M gene mRNAs at 0 h.p.i. and no M2 transcripts; while a total of 47 M gene mRNAs with M2 transcripts accounted for only 8.5% at 16 h.p.i. The above results indicates that H1N1 presents impaired splicing efficiency after infection of human CD8^+^ T cells (Figure 4B). We further examined the dynamic changes of M1, M2, NS1 and NEP via RT–PCR using specific primers, in which there was no overlap between spliced and unspliced amplified fragments (Figure 4C). M gene splicing rate maintained extremely low in CD8^+^ T_EM_, which is in stark contrast to A549 cells. There was no significant difference in mRNA ratio of NEP/NS1 between A549 and CD8^+^ T_EM_ after infection (Figure 4D). However, mRNA expression levels of host splicing factors were up-regulated in CD8^+^ T_EM_ after infection, such as *NS1-bp*, *hnRNP K* [26] and *sf2* [18] unexpectedly (Figure 4E), which indicated that there may be other splicing factors mechanism for controlling a more active splicing process of influenza virus in human T cells.

In addition, ISGs (interferon-stimulated genes) are highly effective at controlling and eliminating influenza A virus in host cells. The responses of the interferon system in human T cells post-infection are worth assessing. The network of ISGs was up-regulated at 16 h.p.i. in infected CD8^+^ T_EM_ from single-cell sequencing data, such as *Mx1* (MX dynamin like GTPase 1), *rsad2* (radical S-adenosyl methionine domain containing 2), *adar1* (RNA specific adenosine deaminase 1) and *OAS* (2′-5′-oligoadenylate synthetase) family (Figure 4F). RT–PCR indeed corroborated an increasing *rsad2*, *adar1*, *oas1* and *oas2*. The *rasd2* gene, in particular, was up-regulated 7-fold within 48 h of infection (Figure 4G).

### The effects of H1N1 virus infection on CD8^+^ T_EM_

**Figure 5.**
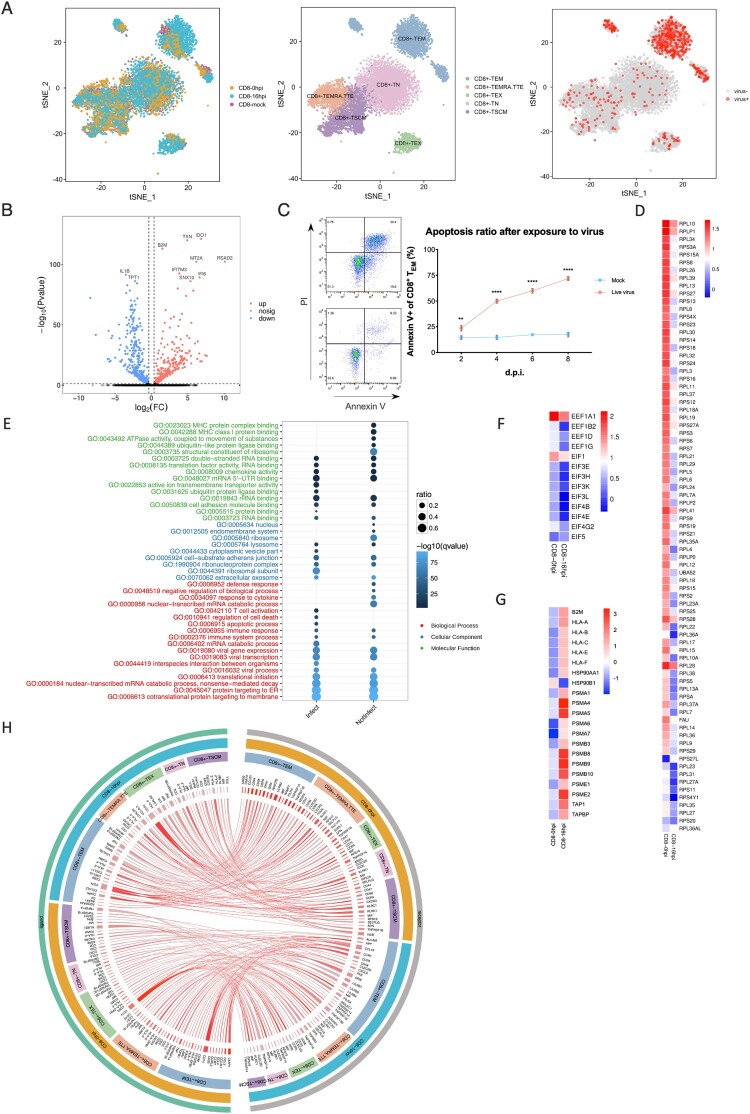
Biological consequences of non-productive infection in infected effector memory CD8^+^ T cells. (A) Left, subsets partition diagram of human CD8^+^ T cells through single-cell sequencing. Right, subpopulation distribution of infected CD8^+^ T cells (red) and bystander cells (grey). (B) Volcano plot of differentially expressed genes in infected CD8^+^ T_EM_ over time. Red, up-regulation; blue, down-regulation. (C) Apoptosis of CD8^+^ T_EM_. Left, cells with annexin V and PI staining on 2 d.p.i. Right, apoptosis ratios of CD8^+^ T_EM_ after exposed to H1N1 over time. Red represents for the group with treatment of live viruses while blue represents for the control group of UV-treated inactive viruses. Results are represented as mean fold change ± SD and statistical significances were analysed using GraphPad Prism 8.0 through two-tailed Student’s *t*-test. *p < 0.05, **p < 0.01, ***p < 0.001, ****p < 0.0001. (D) The heatmap of ribosomal protein-related genes in infected CD8^+^ T_EM_ post-infection over time. (E) GO enrichment pathways change analysis of molecular function (green), cellular component (blue) and biological process (red) among infected and non-infected CD8^+^ T_EM_. (F) The gene change heatmap of eEFs in infected CD8^+^ T_EM_ over time. (G) The gene change heatmap of MHC I-immunoproteasomes in infected CD8^+^ T_EM_ over time. (H) Circos analysis of interactive relationships between membranous receptors and ligands among different CD8^+^ T subsets after exposed to H1N1 over time.

The exposure of primary human T cells to influenza A virus directly *in vitro* did not significantly induce their differentiation through principal component analysis (Figure 5A). And the samples were divided into two groups, one was marked as infected cells with viral UMI (unique molecular identifier) greater than zero, while the other was labelled as bystanders (expose to the virus but not infection). In comparison between infected cells and bystanders, there was only a small amount of genes expression changed post-exposure over 16 h at the same point in time (data not shown). However, respectively for infected cells or bystanders, the expression of a lot of genes has changed post-exposure over 16 h. For instance, *IDO1*, *IFITM3* and *IFI6* were up-regulated while *1L1B* and *TPT1* were down-regulated at 16 h.p.i. in infected CD8^+^ T_EM_ cells (Figure 5B). In addition, upon influenza A virus infection, we identified the significantly decreased abundance of mRNA of host ribosomal proteins in infected human T cells, including small 40S and large 60S subunits (Figure 5D). The abnormal death of total CD4^+^ T, CD8^+^ T and CD8^+^ T_EM_ was detected by annexin V and PI flow staining. We found that as the most susceptible subset, CD8^+^ T_EM_ also had a more severe degree of apoptosis than total CD4^+^ T, CD8^+^ T cells (Figure 5C and S1E).

Then the GO and KEGG pathway enrichment analyses were carried as well as the biological process, molecular function and cellular component (Figure 5E, S1G and S1H). For cellular components, cytosolic parts and ribosomes were enriched. What is noteworthy is that RNA binding, protein binding and ubiquitin-like protein ligase binding pathways were identified to vary greatly in the biological process. What’s more, co-translational proteins targeting to membrane, RNA catabolic process and proteins localization to ER were supposed to have a bigger variance for molecular function (Figure 5E). Significantly, the immunoproteasome-MHC I pathway was up-regulated over time of infection, while the MHC II pathway was down-regulated, which indicated T-host may utilize a ubiquitin-proteasome mechanism to degradant invading influenza viral virions and enhance the function of endogenous antigen presentation (Figure 5G and S1J). Data also showed that eIFs (eukaryotic initiation factors) and eEFs (eukaryotic translation elongation factors) were down-regulated which suggested the translation of host proteins with encumbrance (Figure 5F). The amazing changes of the cytokines network also occurred such as C–C and C-X-C chemokines and corresponding ligands (Figure S1J). Our unbiased and high-throughput screening provided crucial novel insight into host–pathogen interactions of H1N1 infection in primary human T cells.

## Discussion

Human T lymphocytes are the critical components of the adaptive immune system, which provide lifelong protection against pathogens by coordinating immune responses through the whole body effectively. T cells is heterogeneous and mainly comprised of naive T cells, terminally differentiated effector T cells (T_EMRA_), and memory T cells including central memory T cells (T_CM_) in lymph nodes and circulation, effector memory T cells (T_EM_) which circulate in peripheral sites and blood, resident memory T cells (T_RM_) retained in tissues and are regarded as one part of T_EM_ at times. Each of them has specific characterizations in health safeguard. Currently, little is known about the host–pathogen interaction of influenza A virus infection in primary human T cells.

In this study, we demonstrated that CD8^+^ T_EM_ was highly susceptible to H1N1 infection and H1N1-induced apoptosis of T cells. According to the poly(A) positive method for single-cell library construction [27], the virus not only entered into the cell, but also transcribed in the nucleus successfully. What is noticeable is that the staple site of infection in influenza patients is the lung, and previous research has shown that the differential compartmentalization of human T cell subsets was conserved among individuals, with the predominant proportion of CD8^+^ T_EM_ in the lung independent of donor age [28]. In addition, the effector memory subset was identified as one of the key cell phenotypes in cross reactive IFN-γ^+^IL-2^−^CD8^+^ T cells which protected against symptomatic pandemic influenza H1N1 in patients [29]. It implied that CD8^+^ T_EM_ which fighting on the front line in the lung, was very vulnerable to the attack of influenza A virus, and might be damaged with a weakened antiviral function.

Influenza viral RNAemia had been found in some patients with severe pandemic H1N1 with a high amount of viral genome could be detected in the blood during the acute phase of infection [30], while live highly pathogenic H7N9 virus could be isolated from the patient’s plasma as viraemia [31]. Our research results suggested the infected T cells that carried the virus nucleotide and circulated between lung and blood may be another cause to explain. The scientific problem is worthy of note that given the capacity of pandemic H1N1 to invade human T cells, the patients with influenza who develop viral RNAemia may have more serious consequences.

Our previous clinical study has also confirmed that lymphopenia characterized by the reduction of both CD4^+^ and CD8^+^ T cells in peripheral blood is a common feature among influenza-infected patients, and linked to the worse outcome (not yet published). In this research, we revealed that the influenza A virus could directly infect primary human T cells especially CD8^+^ T_EM_ with protective memory function and induce apoptosis in the infected cells. Lymphopenia was also the major among the laboratory abnormalities in acute SARS patients [32], but interestingly, SARS-CoV cannot infect human T cells directly [33]. Accordingly, the potential mechanism of influenza and SARS-CoV induced lymphopenia could be distinguished. Up to now, it has been presumed that lymphopenia detected in cases of respiratory virus diseases might be due to inhibition of thymus for T-cell generation, abnormal cell death, tissue sequestration of lymphocytes, exhaustion, migration or homing from blood [34,35]. In particular, lymphopenia of severe influenza but not SARS patients could be a consequence of a direct infection in T cells and infection-induced apoptosis. It is worth studying the mechanism and treatment of lymphopenia in influenza patients further.

The entry of influenza A viruses into human T cells may dependent on classical sialic acid receptors. The expression of α-2,6-linked sialic acid receptors on the surface of CD8^+^ T_EM_ was higher than other T cells, provided a potential more susceptibility. In addition, the avian influenza virus may also attack human T cells due to the similar expression level of α-2,3-linked sialic acid receptors. Interestingly, several new receptors or attachment factors for entry of influenza A viruses that belong to CD (cluster of differentiation) molecules have been identified recently except non-receptor pathways, such as clathrin-mediated, non-clathrin-mediated and caveolin-mediated endocytosis or micropinocytosis. As a member of the tetraspanins family, CD81 play a central role in organizing endosomal membranes for assisting viral fusion while infecting A549 lung carcinoma cells [36]. The treatment of anti-sialylated glycoprotein CD83 on dendritic cells and macrophages could reduce influenza A virus-lung injury and in mice [37]. Influenza A virus can also use a host adhesion molecule on surface CD66c as a novel receptor that binds to viral NA during entry into A549 and HEK293 cells [38]. Since both of them have immune functions and present on primary human T cells, the exploration of their roles in early infection with the influenza A virus should be taken seriously for some time to come.

Influenza A virus has a unique advantage of a nuclear replication cycle in the host nucleus, unlike the largest number of RNA viruses with a cytoplasmic life cycle such as MERS, SARS-CoV-2 [39], CHIKV, DENV and Zika virus [40]. On the one hand, nuclear replication is helpful to avoid innate immune responses that arise from the monitoring of RIG-I-like helicases [41]; on the other hand, the influenza virus is available to use host splicing apparatus to expand viral gene transcripts. Our research found that human T cells adopted a strategy of controlling viral alternative splicing against invasive viruses, for example, an impaired splicing efficiency of viral M gene was observed in CD8^+^ T_EM_ cells. This was in stark contrast to the previous study of single-cell sequencing in A549 cells, which was a recognized host cell for influenza A viral productive infection [42]. To further mechanism for that, host *hnRNP K* and *ns1-bp* gene were followed with interest which have been identified as the key factors with the co-regulatory activity to mediate viral M gene splicing [26]. But contrary to expectations, the up-regulation of *hnRNP K* and *ns1-bp* gene were found from qPCR (Figure 4E). There may have a more complex splicing mechanism and regulatory networks in nuclei of human T cells. It can be seen that the impaired splicing efficiency is the key speed limit of abortive infection. Importantly, the regulation of viral pre-mRNA splicing is the basic of host-virus interaction, and it may become a novel potential strategy of antiviral intervention.

Interferon signal-inducible immunoproteasomes have a particular catalytic core particle to help antigen presentation on MHC class I efficaciously, and are able to respond to immune and inflammatory stimuli quickly due to its rapid assembly rate [43]. The immunoproteasomes activate CD8^+^ T cells as well as regulates responses of CD8^+^ T cells to epitopes of influenza A virus during infection. Our evidence suggested that the up-regulation of MHC class I-immunoproteasomes may constitute intracellular antiviral activities for degrading exogenous viral proteins and resulted in non-productive infection. What’s more, the ability to receive antigen presentation of endogenous Ag was enhanced while exogenous Ag may weaken.

Another powerful antiviral weapon of the IFN system is ISGs. After the virus invaded human T cells successfully, the host up-regulated a series of ISGs rapidly, especially *rsad2*. *Rsad2* is an excellent antiviral gene with dual functions, it can not only direct block the synthesis of the viral RNA chain by inducing false cytidine triphosphate (CTP) [44], but also inhibits the budding of influenza A virus through interfering lipid rafts on the surface [45]. It can be thought of as “armour” for CD8^+^ T cells facing the complex infectious environment in the lung. It should be pointed out that even if the progeny viruses were not amplified successfully, the abortive infection has caused profound damage to human CD8^+^ T cells, such as shutting down the host protein transcription-translation system (may stalls mRNA translation or reduces the translation capacity), causing intense apoptosis and increasing inflammatory cytokines.

At present, many groups have focused on exploring drug targeting restoration of lymphocyte counts, especially human T cells, and have made many explorations to achieve the effect of antiviral therapy. For example, thymosin α-1 [46], Azvudine [47] and Opaganib [48], have been explored actively for the treatment of SARS-CoV-2 infection. Due to the high proportion in human lungs [28] and significant anti-influenza virus ability at the front, developing methods to increase or stabilize the level of CD8^+^ T_EM_ counts in the lung may improve the immune responses to influenza A virus infection among pandemics. What’s more, the block of viral M splicing may be a key component of CD8^+^ T_EM_-viral response, and it provides potential new targets of antiviral intervention and needs further attention.

Currently, there are still several gaps in understanding of the viral life cycle in primary human T cells and, in particular, the mechanisms by which T cells block replication of influenza A virus. In addition, it is still unclear whether some highly pathogenic avian influenzas can evolve or mutate strategies to overcome the restriction of human T cells. To further seek the cellular factors associated with restriction of pandemic H1N1, understanding their expression in different human T subpopulations that restrict productive viral replication and investigating strategies to induce expression of such host factors in epithelial cells for antiviral treatment to represent significant roads of future research. In summary, herein we demonstrated a novel antiviral strategy of host cells. And this research reveals significant information of pandemic H1N1 infection in primary human T cells. Our results contribute to the understanding of severe lymphopenia, viral spread, high transmissibility and suggest potential targets of defence against influenza A virus.

## Supplementary Material

Supplemental Material
