# Supplementary figures and images for: Pandemic influenza A (H1N1) virus causes abortive infection of primary human T cells
Source: Emerg Microbes Infect. 2022 Apr 25;11(1):1191–204. doi: 10.1080/22221751.2022.2056523 (PMC9045768; doi:10.1080/22221751.2022.2056523)

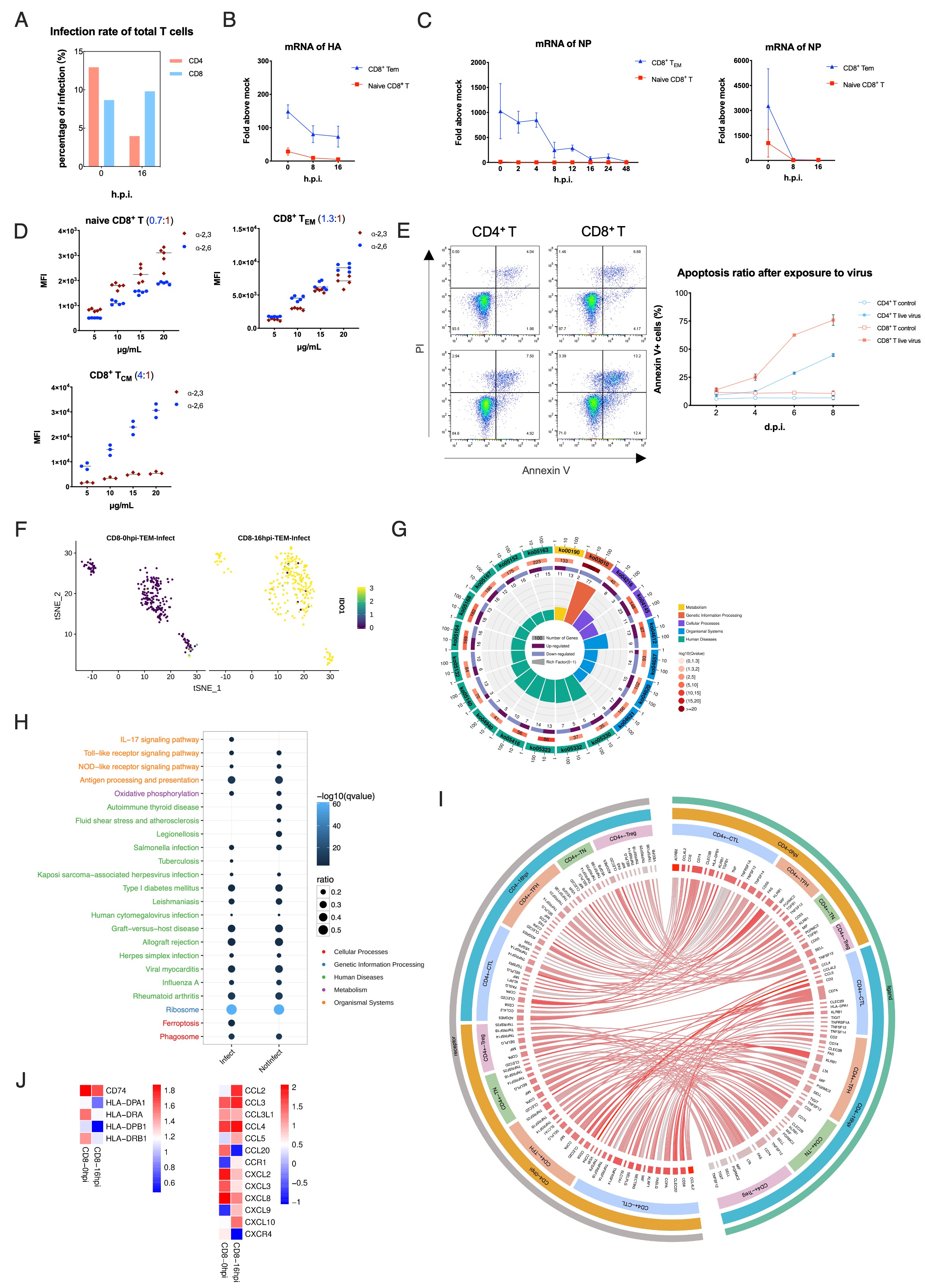

Supplement: Supplemental Material [file TEMI_A_2056523_SM8092.jpg]
